# Age-Related Structural Alterations in Human Amygdala Networks: Reflections on Correlations Between White Matter Structure and Effective Connectivity

**DOI:** 10.3389/fnhum.2019.00214

**Published:** 2019-07-05

**Authors:** Yuhao Jiang, Yin Tian, Zhongyan Wang

**Affiliations:** Bio-information College, ChongQing University of Posts and Telecommunications, ChongQing, China

**Keywords:** white matter structure, effective connectivity, amygdala, fractional anisotropy, aging, emotion

## Abstract

The amygdala, which is involved in human social information processing and socio-emotional response neuronal circuits, is segmented into three subregions that are responsible for perception, affiliation, and aversion. Though there is different functional and effective connectivity (EC) among these networks, age-related structural changes and associations between structure and function within the amygdala remain unclear. Here, we used diffusion tensor imaging (DTI) data (106 participants) to investigate age-related structural changes in fractional anisotropy (FA) of amygdalar subregions. We also examined the relationship between FA and EC within the subregions. We found that the FA of the amygdalar subregions exhibited inverted-U-shape trends with age. Moreover, over the human lifespan, there were negative correlations between the FA of the right ventrolateral amygdala (VLA.R) and the Granger-based EC (GC) of VLA.R → perception network (PerN), the FA of the VLA.R and the GC of the net flow from VLA.R → PerN, and the FA of the left dorsal amygdala (DorA.L) and the GC of the aversion network (AveN). Conversely, there was a positive correlation between the FA of the DorA.L and the GC of the net flow from DorA.L → AveN. Our results suggest that age-related changes in the function of the brain are constrained by the underlying white matter architectures, while the functional information flow changes influence white matter structure. This work increases our understanding of the neuronal mechanisms in the maturation and aging process.

## Introduction

The amygdala is involved in human social information processing and socio-emotional response neuronal circuits, and it plays an important role in dynamic emotional changes during human life. As a component of the “social brain,” the amygdala is a subcortical structure (Adolphs, 2009). Along with other related brain regions, including the fusiform gyrus (FFA), lateral orbitofrontal cortex (lOFC), anterior cingulate cortex (ACC), ventromedial prefrontal cortex (vmPFC), and rostral superior temporal sulcus (rSTS; Bickart et al., 2014; Wang et al., 2014; Kruschwitz et al., 2015; Ebisch et al., 2016; Di et al., 2017), it contributes to adapting the integrative processing of social information that underlies the awareness of other individuals’ affective experiences in complex social perception (Jiang et al., 2019). Many neurodevelopmental and neurodegenerative disorders are related to abnormal amygdalar function (Ledoux, 1996). Despite the crucial role of this region, little is known about age-related effects on human amygdalar subregions, which are identified by structure and function.

Myriad studies focus on functional connectivity (FC) between the amygdala and its related networks based on the NKI-RS database^1^. Researchers explored the FC of the entire amygdala and other regions/networks (e.g., attentional networks) with age, including the visual cortex in healthy adults and multiple cortical networks in adulthood (Oler et al., 2012; Laird et al., 2013; He et al., 2016; Mather, 2016). Researchers also noted different FC trajectories across the human lifespan: aging-relevant inverted-U-shaped trajectory on network properties (Zhao et al., 2015), a strong correlation between functional hubs in adulthood contrast to childhood (Uddin et al., 2011), and a linear decline and an inverted-U-shaped trajectory for the rich club during human life (Cao et al., 2014). These findings indicate that amygdala plays a crucial role in social information processing and emotional control despite variable age-related FC trajectories.

According to different cognitive and emotional representations in social information processing, the amygdala, which contains multiple nuclei, is segmented into three subregions. The ventrolateral amygdala (VLA) is involved in the social perception network (PerN), which includes the lOFC, FFA, rostral STS, ventromedial temporal cortex, temporal pole, and subgenual ACC. The medial amygdala (MedA) is related to the affiliation network (AffN), which includes the vmPFC, ventral medial striatum of the nucleus accumbens, ventromedial hypothalamus, adjoining subgenual and rACC, dorsomedial temporal pole, and medial temporal lobe. The dorsal amygdala (DorA) is associated with the aversion network (AveN), which includes the caudal ACC, ventrolateral striatum, anterior insula, somatosensory operculum, caudolateral hypothalamus, thalamus, and brainstem (Adolphs et al., 1994; Price, 2007; Höistad and Barbas, 2008; Sallet et al., 2011; Hart et al., 2014; Von Der Heide et al., 2014; Ball et al., 2015; Rolls, 2015; Rutishauser et al., 2015; Ferenczi et al., 2016; Hampton et al., 2016; Jiang et al., 2019). Moreover, FC, which represents the temporal coherence between spatially remote neurophysiological events, does not reveal a potential information flow direction between regions/networks. Therefore, effective connectivity (EC) based on Granger-causality analysis (GCA) between amygdalar subregions and their related networks helps investigate age-related information flow trends. Our recent work reported different information flow trajectories across human life, including an increased VLA → PerN trend with aging, a U-shaped PerN → VLA trend, an inverted-U-shaped trajectory for the causal influence net flow from the VLA to PerN, a U-shaped MedA → AffN trend, an increased AveN → DorA trend, and decreased net flow from the DorA to AveN (Jiang et al., 2019).

Converging evidence agrees that changes in whole amygdalar functions from development to maturity are apparent from childhood to adolescence (Thomas et al., 2001; Monk et al., 2003; Killgore and Yurgelun-Todd, 2006) and degeneration with aging (Mather, 2016). However, age-related structural changes are poorly defined. While several studies report that amygdalar volume does not change in the development (Caviness et al., 1996; Lebel et al., 2008), others observe differential age-related changes with gender differences (Gerber et al., 2009). Some researchers found that amygdalar volume decreases as age increases (Ziegler et al., 2012; Fjell et al., 2013; Pfefferbaum et al., 2013) and differs depending on gender, age range, and clinical conditions (Ruigrok et al., 2014; Zanchi et al., 2017). A recent study demonstrated that all amygdalar subregions are negatively correlated with age from 18 to 69 years and show subarea-specific trajectories (Kurth et al., 2019). Comparatively, two previous studies did not observe decreased longitudinal amygdalar volume (Frodl et al., 2008; Cherbuin et al., 2011).

Given the conflicting findings on amygdalar structure and volume changes, along with the consistent functional alterations, it is possible that amygdalar connectivity patterns may represent structural changes that underlie functional modulations (Saygin et al., 2015; Mather, 2016). For example, a larger amygdala is associated with more stressful influence (Callaghan and Tottenham, 2016), and decreased amygdalar gray matter density occurs after a pressure decline program (McEwen et al., 2016). Social network size also affects amygdalar volume during human life. Moreover, amygdalar intrinsic integrity is affected by connections with other brain regions, including age-dependent changes in amygdala-based network FC (Tomasi and Volkow, 2012). Furthermore, for young adults, the structural integrity of a white matter tract, which includes the uncinate fasciculus with connections between the amygdala and ventrolateral PFC, is related to trait anxiety scores. Lower anxiety scores may be associated with better structural integrity, but the opposite relationship was also reported (De Witte and Mueller, 2017). The white matter intensity of uncinate fasciculus is decreased with aging (Taylor et al., 2007). However, the age-dependent effects of anxiety on structure are not apparent (Walf and Frye, 2006; Qin et al., 2014). Previous studies implicate the structure connection between the amygdala and vmPFC and reveal negatively emotional regulation in early adulthood but positively emotional modulation in late adulthood (Scheibe and Carstensen, 2010; Kim et al., 2011).

Convergent evidence suggests that anatomical architecture upholds functional interactions in the brain and functional activity reflects its underlying structure (Greicius et al., 2009; Leong et al., 2016). However, little is known about age-dependent structural integrity and structure–function correlation changes in human amygdalar subregions and their networks, particularly with respect to socio-related processing. Usually, diffusion tensor imaging (DTI) is very sensitive to changes in microstructural white matter (Salat et al., 2005). Fractional anisotropy (FA) is commonly employed to explore the structural integrity of tissue microstructure on white matter, and FA magnitude may represent how effectively signal is transmitted in an anatomical pathway and could implicate a functional activation to a stimulus (Warbrick et al., 2017). Thus, decreased FA is considered an indicator of increased diffusion; in other words, decreased FA demonstrates less organization (Davis and Moayedi, 2013). Moreover, FA might be involved with the degree of myelination, white matter fiber tract organization, and axonal branching (Beaulieu, 2002).

In this study, we first investigated the integrity of amygdalar subregion white matter tracts (based on FA) to obtain age-related structural changes. Next, based on our previous age-related EC study in the amygdala (Jiang et al., 2019), the FA and the EC correlations between amygdalar subregions and their networks were further analyzed to understand how age-related brain changes influence social information processes and emotional regulation.

## Materials and Methods

### Ethics Statement

The data set was obtained from the Nathan Kline Institute/Rockland Sample (NKI–RS^1^, Nooner et al., 2012). All data acquisition and sharing were approved by the institutional review board of the Nathan Kline Institute. All participants provided written informed consent. For child participants who were unable to provide informed consent, written informed consent was obtained from their legal guardians.

### Participants

The sample consisted of 106 participants (46 females, age range 10–85 years, *M* = 38.8 years; 60 males, age range 7–81 years, *M* = 38.3 years; age ≤ 15, *n* = 11; age 16–25, *n* = 32; age 26–45, *n* = 24; age 46–65, *n* = 23; age ≥ 65, *n* = 16). All participants are right-handed and not diagnosed with any mental abnormalities.

### Data Acquisition

All three-dimensional T1, T2, and DTI imaging data were obtained with a 3-T Siemens Trio scanner. High-resolution T1-weighted structural data were obtained with magnetization-prepared rapid gradient echo sequence (TR = 2,500 ms, TE = 3.5 ms, flip angle = 8°, thickness = 1.0 mm, slices = 192, in-plane matrix = 256 × 256, field of view = 256 mm). T2-weighted functional data were obtained with a single-shot, gradient-recalled echo-planar sequence (TR = 2,500 ms, TE = 30 ms, flip angle = 80°, field of view = 216 mm, in-plane matrix = 64 × 64, slices = 38, thickness = 3.0 mm, volumes = 260). DTI was conducted with sequence parameters (TR = 10,000 ms, TE = 91 ms, field of view = 256 mm, slices = 58), non-diffusion-weighted images (*b* = 0 s/mm^2^), and 64-direction diffusion-weighted images (*b* = 1,000 s/mm^2^).

### Functional Magnetic Resonance Imaging Data Processing

All GC analysis was based on our recent work (Jiang et al., 2019), including the following sections on functional magnetic resonance imaging (fMRI) preprocessing, identifying networks associated with each amygdalar subregion, and EC based on GCA. The details are also shown in the Supplementary Information.

### DTI Preprocessing

DTI data were preprocessed using a sequence of standard steps based on voxel-based analysis (VBA), and processing was conducted with the SPM8^2^. This procedure involved: (1) correction for eddy-current-induced distortion and head movement using affine registration to the imaging volume (*b* = 0) with no diffusion weighting; (2) evaluating diffusion tensor metrics and FA on each voxel; (3) registering all the individual FA images to the FA template in the MNI space; and (4) spatial smoothing using a 6-mm full-width at half-maximum Gaussian kernel for high signal-to-noise ratio, and resampling to 2 × 2 × 2 mm^3^ voxels.

### Identifying Amygdalar Subregions

In order to identify the emotional networks (PerN, AffN, and AveN) associated with amygdalar subregions, three seed regions of interest (ROIs) in lOFC, vmPFC, and cACC were created (Bickart et al., 2012). Subsequently, we calculated Pearson’s correlation coefficient (*r*) between each ROI and the left and right hemispheres, respectively. The correlation results were transformed to *z* (*r*) maps using Fisher’s *r*-to-*z* transformation, and the whole amygdala was depicted based on Harvard–Oxford Subcortical Structural Atlas probabilistic maps with thresholds at 50%. After excluding gender and age as covariates, we conducted one-sample *t*-tests [*p* < 0.05 with familywise error (FWE) correction] on *z* (*r*)-transformed maps. Finally, we identified amygdalar subregions by identifying which voxels in amygdala showed the strongest connectivity with each ROI on the statistically salient brain regions using paired *t*-tests (*p* < 0.05, FWE corrected; Jiang et al., 2019).

### Amygdalar Subregion FA

We created spherical seed ROIs for each left and right hemispheric amygdalar subregion (VLA, MedA, and DorA). We then extracted the mean FA values for each constructed ROI from the segmented map.

### One-Sample *t*-Test

Within-group analyses of the left and right hemispheric VLA, MedA, and DorA FA were conducted using one-sample *t*-tests [*p* < 0.05, false discovery rate (FDR) corrected].

### Age-Related Generalized Linear Model

We built an age-related generalized linear model (GLM) to explore the FA alterations in left and right amygdalar subregions across life. Next, multiple linear regression equations for the GLM were constructed with gender as a covariate and with age and age^2^ as predictors. Therefore, the model can be written as:

$$Y=a_{0}+a_{1}\times age+a_{2}\times sex$$

(1) $$Y=a_{0}+a_{1}\times age+a_{2}\times age^{2}+a_{3}\times sex$$

where *Y* denotes the fitted value and *a*_0_–*a*_3_ denote regression coefficients. The prediction model was based on Akaike’s information criterion (Cavanaugh, 1997), and we conducted one-sample *t*-tests on predictor variables.

#### Relationship Between EC and Structure

We calculated the Pearson’s correlation coefficient between the VBA-based FA maps and GCA-based GC maps as mentioned above for each hemispheric amygdalar subregion (*p* < 0.05, FDR corrected).

## Results

### Parcellation of the Amygdala and Associated Social Networks

**Figure 1:**
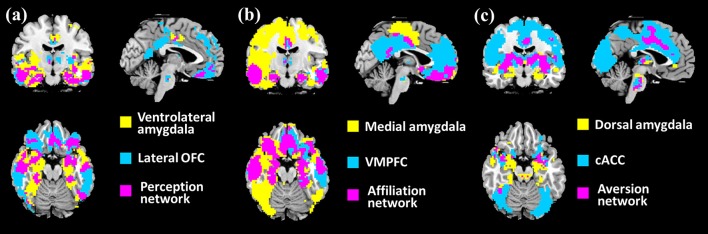
Identification of the associated networks through overlapping between each amygdala subregion-derived binary functional connectivity (FC) map (threshold at *p* < 10^−5^) and homologous regions of interest (ROIs)-derived binary FC map (threshold at *p* < 10^−5^). **(A)** The perception network (PerN) that connected to ventrolateral amygdala (VLA) is delineated by intersection of the VLA-derived binary map and the lateral orbitofrontal cortex (lOFC)-derived binary map. **(B)** The AffN that connected to medial amygdala (MedA) is identified by the intersection between the MedA-derived binary map and the ventromedial prefrontal cortex (vmPFC)-derived binary map. **(C)** The aversion network (AveN) that connected to dorsal amygdala (DorA) is defined by the intersection of the DorA-derived binary map and the cACC-derived binary map. The yellow areas denote the brain regions functionally connected to each amygdala subregion. The blue areas demonstrate the brain regions functionally connected to each ROI seed region. The purple regions represent the amygdala subregion-related networks.

Through the intersection maps between each amygdalar subregion-derived binary FC map and homologous ROI-derived binary FC map, we identified the amygdalar subregion (see Supplementary Figure S1, Supplementary Table S1 of the Supplementary Information) associated networks (PerN, AffN, and AveN), as shown in Figure 1 (also see Supplementary Figure S2 of the Supplementary Information).

**Figure 2:**
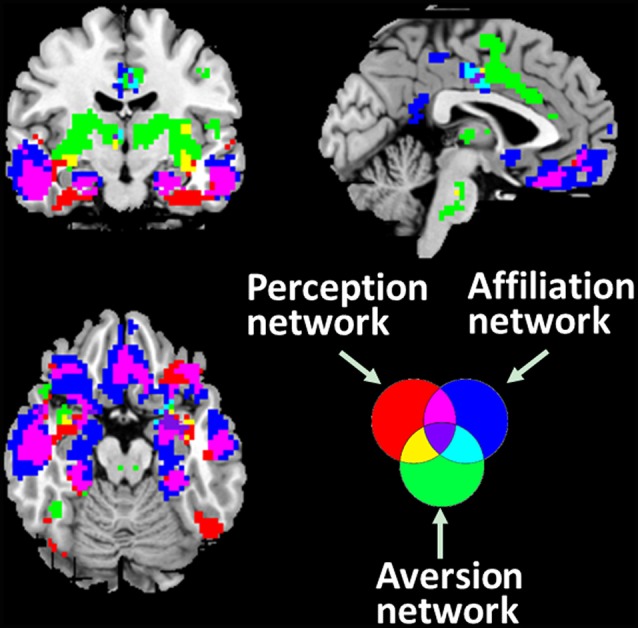
Three distinct corticolimbic networks associated with amygdala subregions. It is noted that some areas are responsible for multiple functions. The red areas denote the PerN, the green areas represent AveN, the deep blue regions refer to AffN, the yellow areas denote the overlapping areas between PerN and AveN, light blue represents the intersection areas of AveN and AffN, purple denotes the overlapping areas between PerN and AffN, and the dark purple areas denote the intersection regions of PerN, AveN, and AffN (threshold at *p* < 10^−5^).

The spatial patterns that depict the brain regions connected with associated networks are shown in Figure 2. The PerN refers to areas including the lOFC, FFA, rSTS, ventromedial temporal cortex, temporal pole, and subgenual ACC. The AffN is associated with the vmPFC, ventral medial striatum of the nucleus accumbens, ventromedial hypothalamus, adjoining subgenual and rACC, dorsomedial temporal pole, and medial temporal lobe. The AveN connects the caudal ACC, ventrolateral striatum, anterior insula, somatosensory operculum, caudolateral hypothalamus, thalamus, and brainstem (Cicchetti and Dawson, 2002; Bickart et al., 2012; van den Heuvel et al., 2015). Although these networks are responsible for different cognitive functions, we observed some anatomically overlapping regions (Figure 2).

### Age-Related FA Changes in Amygdala-Based Networks

**Figure 3:**
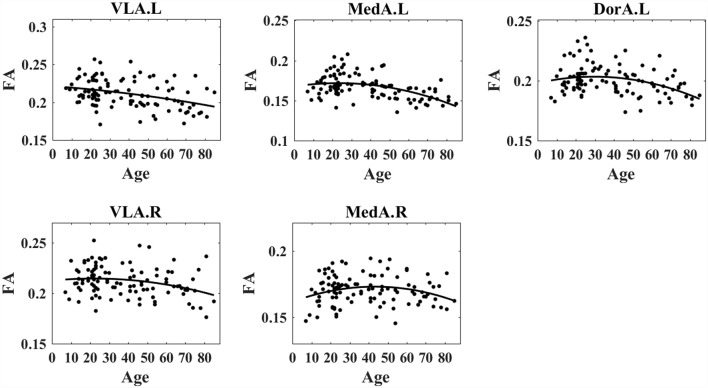
Nonlinear trends of fractional anisotropy (FA) in amygdala subregions with age. The results demonstrated that the FA degree in the left VLA, left MedA, left DorA, right VLA, and right Med demonstrated an inverted-U trend with age.

We obtained specific patterns that described age-related changes in mean amygdalar subregion FA degree vs. age (Figure 3). Considering the wide age distribution (7–85 years), we tested nonlinear trends with a quadratic age function [see Equation (1) in “Materials and Methods” section]. The FA exhibited increased trends during early ages and decreased trends at later ages, i.e., the inverted-U shape with aging. The left VLA FA exhibited a significant quadratic age effect (*t* = −0.37, *p* = 0.0002, *R*^2^ = 0.13) and demonstrated an age-related inverted-U trajectory. For the right VLA FA, the quadratic term was significant (*t* = −1.14, *p* = 0.001, *R*^2^ = 0.16) and showed an inverted-U trend with age. The left MedA FA presented a significant quadratic age effect (*t* = −2.27, *p* = 0.016, *R*^2^ = 0.05) and demonstrated an age-related inverted-U trend. The right MedA FA exhibited a significant quadratic age effect (*t* = −2.20, *p* = 0.01, *R*^2^ = 0.06) and demonstrated an age-related inverted-U trend. The left DorA FA exhibited a significant quadratic age term (*t* = −2.06, *p* = 0.04, *R*^2^ = 0.12) and showed an inverted-U trend with aging. However, there was no significant correlation between right DorA and age. Table 1 presents statistical regression coefficients and the quadratic fitting curve.

**Table 1:** Statistical parameters of age-related FA within amygdala subregions.

FA	*T*	*p*	*R*^2^	Fitting curve
Left hemisphere
VLA.L	−0.37	0.0002	0.13	*y* = −1.6229e^−6^ *age*^2^ + 0.00018 *age* + 0.22125
MedA.L	−2.27	0.016	0.05	*y* = −7.6025e^−6^ *age*^2^ + 0.00035 *age* + 0.16762
DorA.L	−2.06	0.04	0.12	*y* = −6.0714e^−6^ *age*^2^ + 0.00036 *age* + 0.19803
Right hemisphere				
VLA.R	−1.14	0.0001	0.16	*y* = −4.1661e^−6^ *age*^2^ + 0.00018 *age* + 0.21259
MedA.R	−2.20	0.01	0.06	*y* = −6.0717e^−6^ *age*^2^ + 0.00052 *age* + 0.16157

*Note: FA denotes fractional anisotropy, R^2^ represents the curve fitting degree of the age-related quadratic fitting curve*.

### Correlation Between EC and Structure

**Figure 4:**
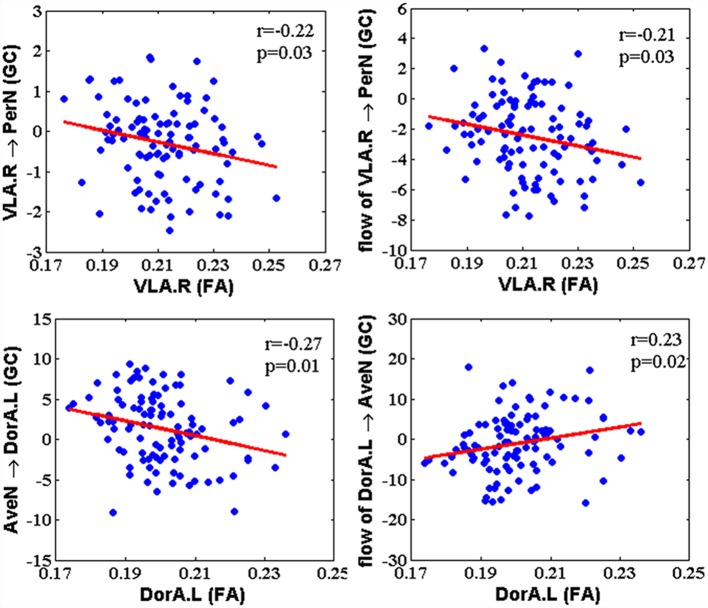
The correlation between Granger-causality (GC) interaction and FA within amygdala subregions. The GC of VLA → PerN and net flow from VLA to PerN were negatively correlated with FA of the right VLA. The GC of AveN → left DorA showed a negative correlation with FA of the left DorA, whereas net flow from left DorA to AveN was positively correlated with FA of the left DorA.

After calculating the EC between amygdalar subregions and their related networks based on GCA, we acquired the correlation between the GC and FA for hemispheric amygdalar subregions (Figure 4; *p* < 0.05, FDR corrected). The GC influence from the right VLA (VLA.R) to PerN was negatively correlated with the VLA.R FA (*r* = −0.22, *p* = 0.03). The GC net flow from the VLA.R to PerN was also negatively related with the VLA.R FA (*r* = −0.21, *p* = 0.03). For GC, the AveN influence to left DorA (DorA.L) was negatively correlated with the DorA.L FA (*r* = −0.27, *p* = 0.01). Regarding GC, the net flow from the DorA.L to AveN was positively correlated with the DorA.L FA (*r* = 0.23, *p* = 0.02). None of the other hemispheric amygdalar subregions exhibited significant correlations with their related networks.

We used slipping age windows to clarify the age effect on the correlation between GC and FA, since the number of subjects for each age stage was the limiting factor in this study. The GC–FA correlation revealed an age-related inverted-U shape with VLA.R → PerN, the flow of the VLA.R → PerN, and AveN → DorA.L, and an age-related U shape with the flow of DorA.L → AveN (Supplementary Figure S3, Supplementary Table S2). Although the GC–FA relationship was not significant for some age stages, it could provide a reference for deeper understanding of age-related structure–function correlations.

## Discussion

As a crucial constituent of the limbic system, the amygdala represents an anatomical and functional substrate that synthesizes information from multiple emotions. In this work, we acquired lifetime patterns that describe structural changes in distinct amygdalar subregions based on an age-related GLM. We investigated the correlation between EC and amygdalar structure.

### Age-Dependent Amygdalar Structural Changes

Although research on age-related amygdalar structural alterations is not as abundant as that on FC, changes in its structural architecture are also important for understanding emotional behavior and processing with aging.

Consistent with the prior finding of widespread decreases in FA values in older compared to younger people (Burzynska et al., 2010; Evers et al., 2012), we observed that FA values showed inverted-U-shape trends in VLA.L, MedA.L, DorA.L, VLA.R, and MedA.R during life (Figure 3). Our results confirm and extend previous findings of lower overall connectivity, and lower local efficiency in older adults using probabilistic tractography (Gong et al., 2009), as well as lower rich club organization in older adults using deterministic fiber tractography (Zhao et al., 2015). Additionally, lower FA values suggest a decline in information transmission efficiency (Rus et al., 2017). A possible explanation for these results is that structural connections are pruned or eliminated during aging (Saygin et al., 2011). More specifically, the white matter tracts with respect to PerN-, AffN-, and AveN-specific functions are pruned with age, and thus the function of the amygdala becomes more targeted. Further, the eliminated structural connections might stem from neurophysiological changes, i.e., a decreasing degree of myelination, axonal branching, and fiber tract number (Zatorre et al., 2012).

Prior structural research revealed that amygdalar volume is positively correlated with stress and experience; amygdalar gray matter density is reduced after a decline in stress (Hölzel et al., 2010). Additionally, there is a positive association between amygdalar volume and social network complexity in adults (Bickart et al., 2011). Consequently, these reported results indirectly support the explanation that a reduction in neural functional specificity can be attributed to neurophysiological changes in aging.

Converging evidence suggests that the left amygdala exerts more influence on cognitive and intentional emotion compared to the right amygdala. On the other hand, the right amygdala participates more in social and instinctive emotional reactions (Gläscher and Adolphs, 2003; Liu et al., 2015). This finding might clarify our inconsistent FA changes in the left and right amygdala. The inverted-U trend of FA changes in the right MedA may result from a specific pattern of prosocial-behavior-related brain activity, which initially decreases during pubertal development and subsequently increases in older adults.

### Age-Related EC–FA Relationships for Amygdalar-Subregion-Based Networks

Previous research reported lower intra-network and higher inter-network FC in older compared to younger adults. Further, the neural pattern is not constrained to a specific network but generalizable across systems (Bullmore and Sporns, 2009; Chan et al., 2014; Grady et al., 2016). Intriguingly, Zimmermann et al. (2016) reported that intra-network structural connectivity in hub regions is higher in older compared to younger adults. This contradiction between FC and structural connectivity was consistent with our findings of the EC–FA associations between the VLA and DorA. We found that the GC of the VLA → PerN, the GC net flow of VLA → PerN, and the GC of the AveN→ DorA were negatively correlated with the FA in corresponding amygdalar subregions. Conversely, the GC net flow from the DorA to AveN was positively correlated with FA (Figure 4). These results demonstrate that the EC and FA within amygdalar subregions influence each other during life.

The negative relationships between the GC and the FA during life indicate that the increased FA on structure is associated with decreased EC on function. We observed increased FA with development and decreased FA with aging when using a nonlinear model (Figure 3). Additionally, some GC–FA couples exhibited inverted-U shapes with age (Supplementary Figure S3). In other words, during development, amygdalar white matter tract structural integrity increased, but the VLA.R → PerN and AveN → DorA.L information flows declined. These findings indicate that inter-network information transfer is weak during development. The weaker transfer in children and young adults could help focus on the formation of intra-network connections with specific functions. During aging, the EC–FA correlation for the white matter degrades, but there is stronger intra-network information flow. These findings suggest that amygdalar structural features become sparser and more specific, and the VLA → PerN specificity leads to increased information flow output with aging. These results also demonstrate the VLA role in top-down regulation mechanisms that mediate social perceptual behaviors. An individual’s social perception performance weakly changes with aging. The DorA EC–FA relationship indicates increased AveN → DorA information flow and reduced intra-module white matter structure in old age. Similar to the VLA EC–FA correlation, the AveN → DorA leads to increased input signal with age.

The EC–FA relationship for the DorA → AveN net flow exhibits an age-dependent U-shaped trend. The EC and FA changes present a consistency trend, i.e., the age-dependent structural FA increase is associated with the elevated functional EC during development and the decreased FA is associated with decreased EC during aging. Thus, the lower net DorA → AveN information flow underscores the fact that older individuals exhibit better emotional control when faced with negative environmental stimuli (Birditt, 2014; Dolcos et al., 2014).

Prior behavioral and neuroimaging research demonstrated that individuals exhibit more attachment behaviors in childhood, but affiliative behaviors are reduced in puberty. Subsequently, individuals then exhibit more prosocial behaviors (Sze et al., 2012; Swartz et al., 2014). Notably, there was no statistically significant correlation between the GC and FA within the MedA. A possible interpretation for this result is that an individual’s affiliative behaviors are influenced by blended factors such as peer competition, social relation, educational status, esteem, and social equity (Schlund and Cataldo, 2010; Silke et al., 2018). Thus, for different individuals, the AffN → MedA input signals differ greatly, and the relationship between GC and FA within the MedA is not statistically different.

Taken together, the age-related changes in white matter structural patterns revealed by our findings provide an anatomical basis for understanding the changes in social information processing and emotional behaviors by amygdalar-subregion-relevant networks during life. The resultant network-level FA–EC relationships highlight that analysis of directed information flow might promote structural changes in amygdalar subregions during aging. Contrarily, sparse structural architecture could uphold the functional specificities of emotional behaviors.

## Limitations

There are two noteworthy limitations that should be considered when explaining our results. First, we utilized a cross-sectional design, which is faster and more cost-effective compared to longitudinal designs. However, the sample size was small for the development stage (younger ages) and was not very well distributed across all age ranges. The number and period might affect the explanation of results when applying cross-sectional designs to age-related research (Lindenberger et al., 2011). Longitudinal studies are important for validating the observed age-related alterations in white matter structure from cross-sectional studies, and they might help to interpret the causal correlations between function and structure. Second, we investigated white matter structural changes in amygdalar subregions across the lifespan, but the participant ages made it difficult to determine the timing of specific connectivity changes in amygdalar subregions. It is likely that more longitudinal research that spans a broader age range and multiple age points are crucial to examine structural and FC changes. Additionally, the lack of the nuisance variable such as education years of participants in the NKI-RS database could limit our analysis on the age-related alteration in socio-emotional networks.

## Conclusion

In the current study, we examined the three segmented amygdalar subregions from the angles of social information processing and emotional control. We also explored age-related changes in white matter integrity of amygdalar subregions. Interestingly, we discovered an age-related effect in the amygdala *via* a correlation between structure and function. The results demonstrated both consistency and discrepancy with regard to structural and functional changes. While structural architecture changes influence emotional functions, information flow also impacts amygdalar subregion structural alterations. This study could provide a theoretical foundation for researchers to understand the process of brain maturation and aging.

## Author Contributions

YT: experiments design, conception and writing. YT, ZW and YJ: data analysis and the draft writing, discussed the design, data analysis and results. YT and YJ: contributed reagents, materials and analysis tools.

## Conflict of Interest Statement

The authors declare that the research was conducted in the absence of any commercial or financial relationships that could be construed as a potential conflict of interest.

## Footnotes

^1^http://fcon_1000.projects.nitrc.org/indi/pro/nki.html

^2^https://www.fil.ion.ucl.ac.uk/
