# Supplementary material for: Age-Related Structural Alterations in Human Amygdala Networks: Reflections on Correlations Between White Matter Structure and Effective Connectivity
Source: Front Hum Neurosci. 2019 Jul 5;13:214. doi: 10.3389/fnhum.2019.00214 (PMC6624785; doi:10.3389/fnhum.2019.00214)
Supplement: Supplementary file 1 [file Data_Sheet_1.docx]

**Supplementary Information**

**fMRI preprocessing**

After excluding the first four volumes, a series of defined open-source steps were applied for preprocessing the fMRI data based on SPM12 (http://www.fil.ion.ucl.ac.uk/spm/software/spm12) and REST software (http://restfmri.net/forum/REST_V1.8). This involved 1) slice-timing correction for each voxel's time-series, 2) realignment for correcting rigid body motion (translation ≤ 1.5 mm, rotation ≤ 1.5 degree), 3) normalizing images of each subject to the T1 structural images of individual subjects, then spatial normalizing to the MNI space by EPI template, resampling to 3×3×3 mm^3^ voxels, 4) spatial smoothing based on a half-maximum Gaussian kernel with 5 mm full-width, 5) extracting the signal with frequency range over 0.0078–0.10 Hz, and 6) regressing out sources of 24 variance from data using Friston-24 linear regression using a Friston-24 model, which includes 6 rigid-body head motion parameters, and their first temporal derivatives, and the both items squared. Besides, we also excluded the global, white matter and cerebrospinal fluid signals.

**Identifying networks associated with each amygdala subregion**

To delineate the subregions of amygdala, three seed regions of interest (ROIs) in lOFC, vmPFC, and cACC, were created. In addition, these ROIs interconnect with PerN, AffN, and AveN, respectively ([Price and Drevets, 2010](#_ENREF_36);[Barbas et al., 2011](#_ENREF_1);[Bickart et al., 2012](#_ENREF_5)). Subsequently, Pearson’s correlation coefficient (*r*) was calculated between each node and the left and right hemispheres, respectively. The correlation results were converted to *z* (*r*) maps using Fisher’s *r*-to-*z* transformation, and the whole amygdala was depicted based on Harvard-Oxford Subcortical Structural Atlas probabilistic maps with thresholds at 50%. After excluding gender and age as covariates, we conducted one-sample *t*-tests (p < 0.05 with familywise error correction [FWE]) on *z* (*r*) transformed maps. Last, we identified amygdala subregions by conducting contrast analyses on the statistically salient brain regions using paired *t*-tests (p < 0.05, FWE). In other words, we identified which voxels in amygdala showed the strongest connectivity with each ROI, respectively.

Subsequently, to identify each amygdala subregion associated networks, we created spherical seed ROIs for VLA, MedA, and DorA and constructed FC maps by calculating the Pearson’s correlation coefficient between each amygdala subregion and each hemisphere. Next, we transformed the correlation matrix to *z* (*r*) maps and conducted one sample *t*-tests on transformed *z* (*r*) maps within bilateral VLA, MedA, DorA, lOFC, vmPFC, and cACC (thresholded at p < 10^−5^ and cluster size > 10 voxels). We excluded covariates of gender and age and binarized the FC map with statistical significance. Consequently, PerN, AffN, and AveN can be identified by the intersection map between each amygdala subregion-derived binary map and corresponding ROI-derived binary map, respectively.

**Effective connectivity between amygdala subregions and the associated network**

GCA provides a method to evaluate directed functional influence based on time-series data. We used REST-GCA in the REST toolbox (http://www.restfmri.net/forum/REST_V1.8) to calculate the EC between the reference time series $X_{t}$ of each area (VLA, MedA, and DorA) and each voxel time series $Y_{t}$ of its related network (PerN, AffN, and AveN). Moreover, the regressive equations can be expressed as:

$\begin{matrix} Y_{t}=\sum_{n=1}^{m} \alpha_{n}X_{\left(t-n\right)}+\sum_{n=1}^{m} \beta_{n}Y_{\left(t-n\right)}+\text{C}Z_{t}+E_{t} \\ X_{t}=\sum_{n=1}^{m} \alpha_{n}^{'}Y_{\left(t-n\right)}+\sum_{n=1}^{m} \beta_{n}^{'}X_{\left(t-n\right)}+\text{C}'Z_{t}+E_{t}^{'} \end{matrix}$ (3)

where $\alpha_{n}$ and $\alpha_{n}^{'}$ represent symbol path coefficients, $Z_{t}$ denotes covariates, $E_{t}$ and $E_{t}^{'}$ were residuals, $\beta_{n}$ and $\beta_{n}^{'}$ denote autoregressive coefficients, t denotes time point, and *n* denotes order. Last, we obtained *z*-value GCA maps for all participants based on Fisher’s *r*-to-*z* transformation.

Fig. S1 Three emotional networks associated amygdalar subregions. Each voxel part shows strongest functional connectivity with lOFC, VMPFC and cACC, respectively. The red area denotes VLA, which shows a strongest functional connectivity with lOFC; the blue area denotes MedA, which shows a strongest functional connectivity with VMPFC; the ayan area denotes DorA, which shows a strongest functional connectivity with cACC (citing from Figure 1 of Jiang et al., 2019).

Table S1. Identification of amygdala nuclei. (citing from Table 1 of Jiang et al., 2019)

Fig. S2 The trends of Granger causality between amygdala subregions and associated networks with age (citing from Figure 2 of Jiang et al., 2019).

Fig.S3 The GC-FA couples with age stages.

Table S2 Statistical parameters of the GC-FA couples with age stages.


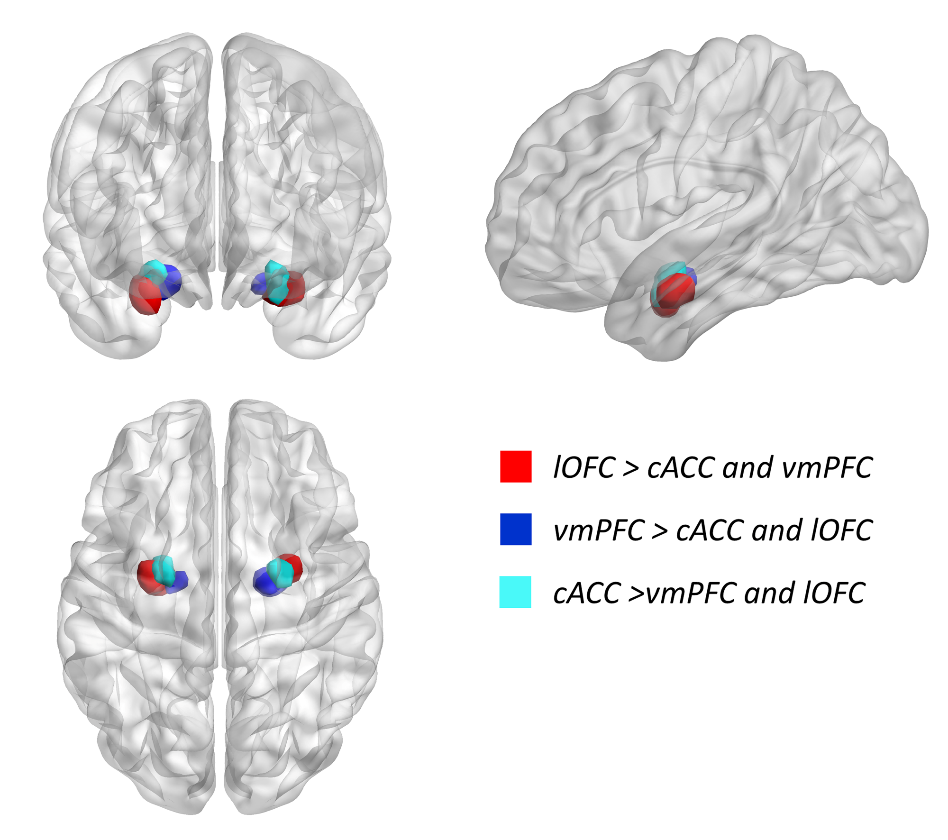


Fig. S1 Three emotional networks associated amygdalar subregions. Each voxel part shows strongest functional connectivity with lOFC, VMPFC and cACC, respectively. The red area denotes VLA, which shows a strongest functional connectivity with lOFC; the blue area denotes MedA, which shows a strongest functional connectivity with VMPFC; the ayan area denotes DorA, which shows a strongest functional connectivity with cACC (citing from Figure 1 of Jiang et al., 2019).

Table S1. Identification of amygdala nuclei. (citing from Table 1 of Jiang et al., 2019)

| **Anatomical** | **Hemisphere** | **Cluster voxels** | **MNI(x,y,z)** |
| --- | --- | --- | --- |
| **Ventrolateral amygdala** | L | 36 | (-27,-3,-21) |
| **Ventrolateral amygdala** | R | 45 | (33,0,-21) |
| **Medial amygdala** | L | 20 | (-15,-8,-18) |
| **Medial amygdala** | R | 26 | (18,-9,-18) |
| **Dorsal amygdala** | L | 18 | (-24,-6,-12) |
| **Dorsal amygdala** | R | 17 | (18,0,-18) |


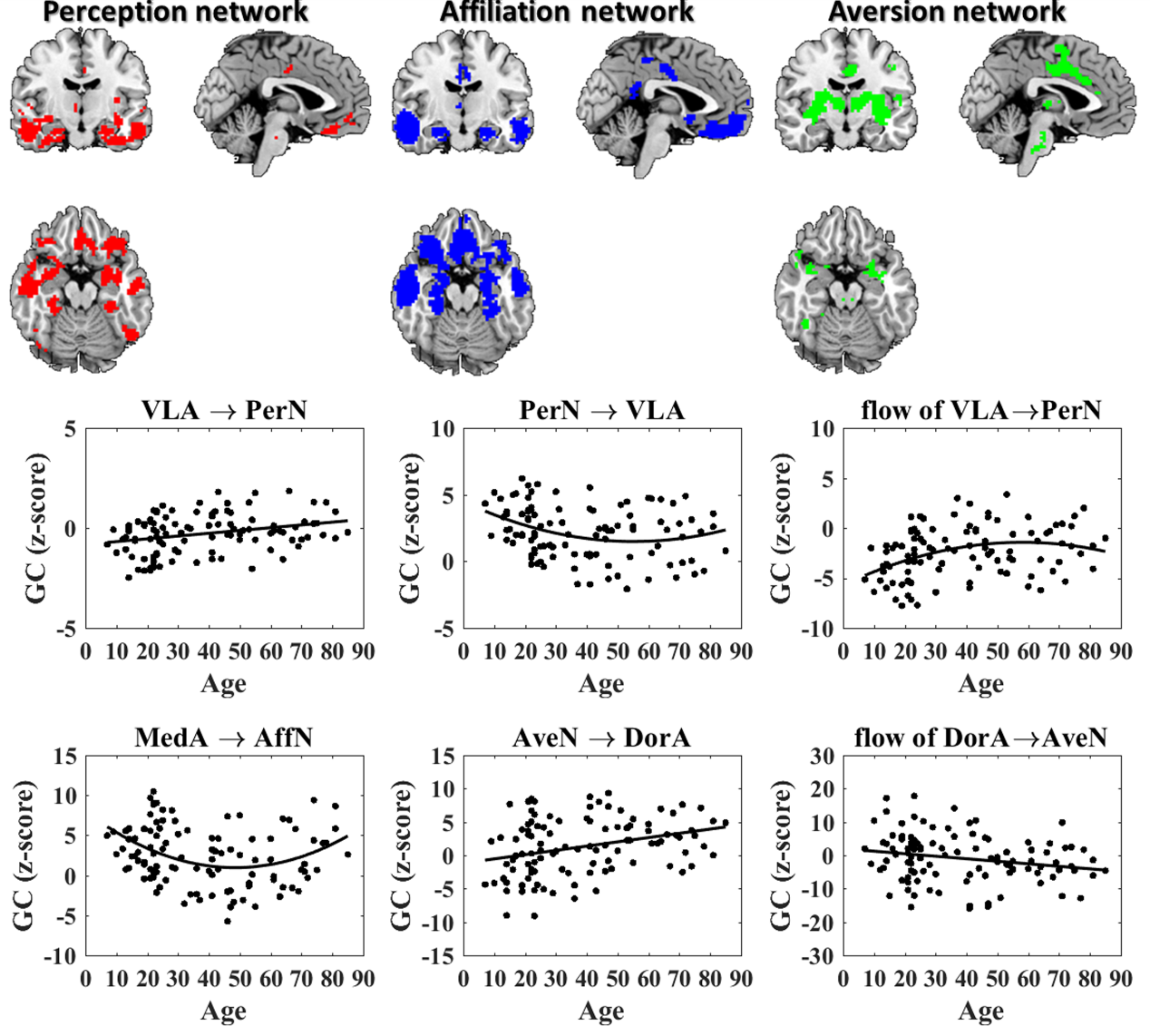


Fig. S2 The trends of Granger causality between amygdala subregions and associated networks with age (citing from Figure 2 of Jiang et al., 2019).


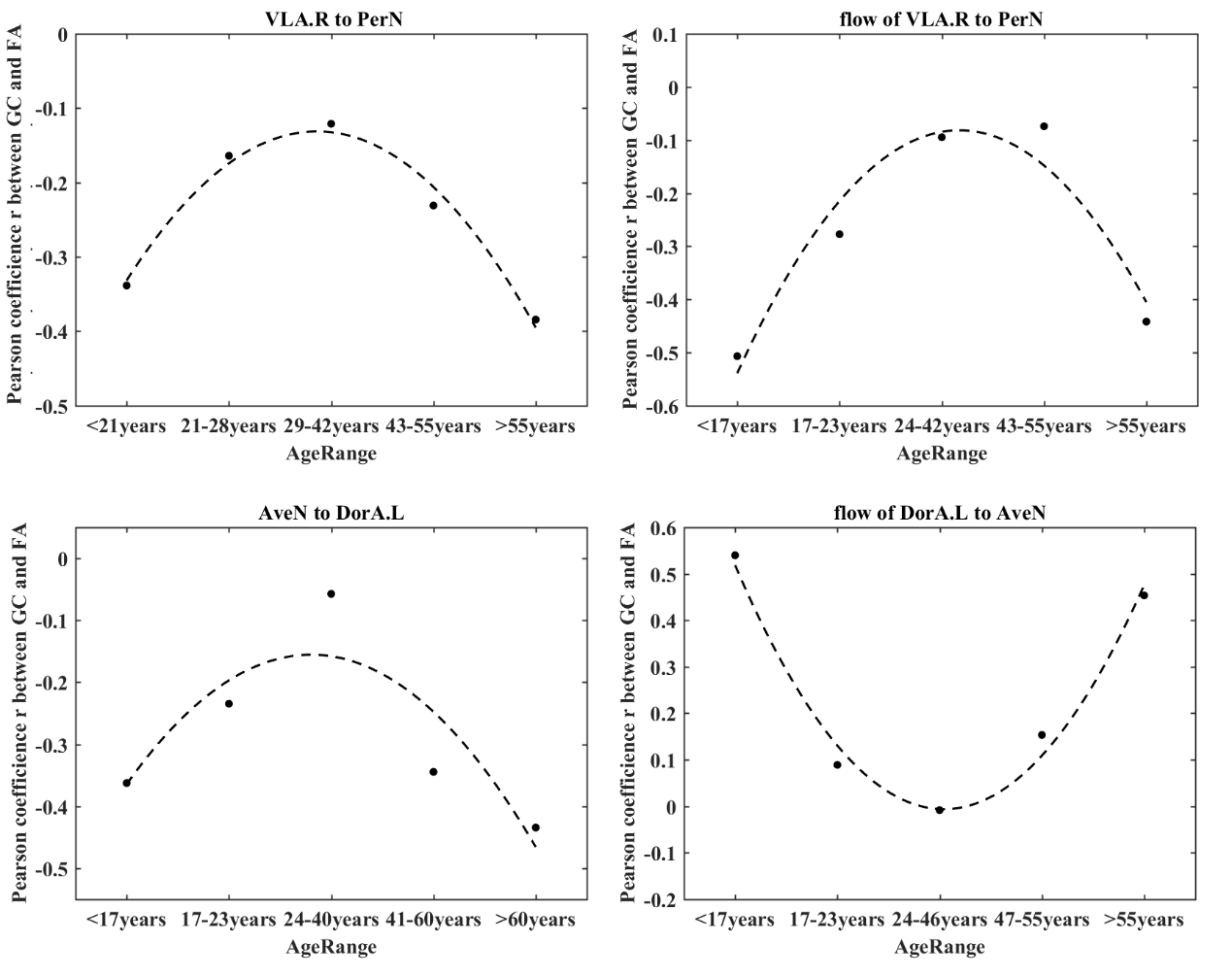


Fig.S3 The GC-FA couples with age stages.

**Table S2** Statistical parameters of the GC-FA couples with age stages.

|  | VLA.R→PerN | |
| --- | --- | --- |
| Age range | p | r |
| <21 | **0.0473★** | -0.3385 |
| 21-28 | 0.4046 | -0.1639 |
| 29-42 | 0.6018 | -0.1209 |
| 43-55 | 0.4946 | -0.2309 |
| >55 | **0.0502☆** | -0.3843 |
|  | Flow of VLA.R→PerN |  |
| Age range | p | r |
| <17 | **0.0531☆** | -0.5069 |
| 17-24 | 0.2814 | -0.2772 |
| 24-42 | 0.6529 | -0.0946 |
| 43-55 | 0.7637 | -0.0738 |
| >55 | **0.0583☆** | -0.4418 |
|  | AveN→DorA.L |  |
| Age range | p | r |
| <17 | **0.0592☆** | -0.3626 |
| 17-23 | 0.2935 | -0.2345 |
| 24-40 | 0.7954 | -0.0572 |
| 41-60 | **0.0784☆** | -0.3446 |
| >60 | **0.0040★★** | -0.4345 |
|  | flow of DorA.L to AveN | |
| Age range | p | r |
| <17 | **0.0467★** | 0.5402 |
| 17-23 | 0.7432 | 0.0890 |
| 24-46 | 0.9592 | -0.0084 |
| 47-55 | 0.6166 | 0.1535 |
| >55 | **0.0522☆** | 0.4538 |

Ps. ★<0.05, ★★<0.01, ☆: marginal significance
